# A Practical Treatise on the Diseases of the Testis, and of the Spermatic Cord and Scrotum

**Published:** 1844-04-01

**Authors:** 


A Practical Treatise on the Diseases of the Testis, and of the Spermatic Cord and Scrotum. With Illustrations. By *T. B. Curling*, Lecturer on Surgery and Assistant Surgeon to the London Hospital, Surgeon to the Jews' Hospital, &c. 8vo. pp. 542. London: Longman and Co. 1843.

Mr. Curling offers the following reasons for selecting diseases of the testis as a subject for investigation, and for publishing a work upon a subject that might seem, if not exhausted, at all events, tolerably well cultivated and understood.

"My attention having been directed in the year 1831, to the subject of the Morbid Anatomy of the Testis, I have since lost no opportunity of studying the pathological changes to which this organ is liable. My inquiries have been much facilitated by a connection formed very early in professional life with a large hospital and with a dispensary, which have supplied me with abundant means of acquiring a practical knowledge of the diseases of this important organ. The result of these investigations having furnished facts which appear of some interest and value in relation to certain affections of the testis but imperfectly understood, and to the treatment generally of the disorders of this part, I have ventured to submit them to the consideration of my professional brethren. In arranging the materials for publication I have endeavoured to give a tolerably complete view of the different diseases of the testis and of the spermatic cord and scrotum, which I have described principally from my own observations. I have at the same time availed myself of the labours of my predecessors; by which, it is hoped, I have not only added a good deal to the value of the work, but have also been able to correct and modify my own views concerning many of the subjects treated of.

"I was unwilling to overload a work which has somewhat exceeded the limits desired with elementary matter, to be found in most anatomical treatises; but as my researches on the structure of the testis have led me to describe certain parts rather differently from other anatomists, and have enabled me to throw some light on the interesting subject of the descent of the testis, I have prefixed a concise, but it is hoped a sufficiently minute account, of the anatomy of the parts in the adult and fœtal states, which, comprising, as it does, the most recent information on this subject, will probably be acceptable to my younger readers." viii.

There is little good done to science by overlaying it with books, more particularly treatises of an elaborate description. If unnecessarily multitiplied they fatigue the reader, disgust the student, and breed a suspicion which damages the whole literature of the profession. Knowledge is progressive, and new works are needed from time to time to record that progress, diffuse an acquaintance with it, and develop the benefits that are to spring from it. If our current literature is below the level of science, whoever, in any department, brings it up to that, deserves every commendation. But if he gratuitously adds a big book to shelves that groan with them already, and, having only half-a-dozen new ideas to communicate, sets to work and ransacks every library to produce a compilation of some hundred pages, which he designates *his* Treatise, commendation is not the due of such a man, but censure of no sparing kind.

Whether Mr. Curling is to be praised or blamed, whether he comes under the former category or the latter, our readers will presently perceive. But this we are bound to observe at starting, that he has always borne a high character with those who know him, for the steady pursuit of professional knowledge, and for no contemptible intellectual capacity.

The Anatomy of the Testis occupies an introductory portion of 48 pages. It may admit of doubt if this was needed. Of what use are systems of anatomy, if an account of the diseases of every organ is to be ushered in by an elaborate description of it? Sir Astley Cooper has set the example, but Sir Astley Cooper had a better excuse than those who follow in the same track—he had the *priority* which they have not. Horace and Sir Fretful Plagiary had the same idea, but Horace unfortunately had the start of the other. Had Sir Astley Cooper, or M. Cruveilhier not written a full account of the testis, the public might have felt indebted to Mr. Curling for his; but, under existing circumstances, it might, perhaps, be spared.

We will, however, run through Mr. C's description of the organ, and notice any new fact or new opinion in it.

1. *Muscularity of the Dartos?*—Our readers may remember enough of anatomy, to be aware that there have been many disputes on the structure of the dartos. Some have stood up for its muscularity—others have looked on it as little better than cellular tissue—and Cruveilhier, one of the latest authorities, regards it as a peculiar contractile sort of tissue, which he names, after it, the *Dartoïd.* Mr. Curling seems an advocate for its muscularity.

"The dartos has recently been carefully examined by Mr. Bowman, who considers it to be muscular, and composed of 'unstriped elementary fibres,' the presence of which, in consequence of the abundant admixture of areolar tissue, has not hitherto been clearly recognised.

"The movements of the scrotum, by the action of the dartos, are conspicuous enough; they are gradual, vermicular, and involuntary. Mr. Bowman states, that since satisfying himself of the existence of the unstriped fibre in the dartos, he has on many occasions detected a very decisive peristaltic action, advancing from one side of the scrotum to the other, and continued for a considerable period. This I have also myself observed. Contraction is excited by cold, and takes place under the influence of fright and during the venereal orgasm: relaxation is caused by heat: galvanism produces no effect." 2.

The microscope is all the fashion just now, and is a sort of judge and jury to a last appeal, on every litigated point. But it is fallible, as experience proves, and we had rather it were supported by other evidence, before we receive its dicta as conclusive. These "unstriped" fibres should be subjected to chemical analysis, and seen by other gentlemen as well as Mr. Bowman. That vermicular movement of the scrotum—noticed by Mr. Curling, must have been also noticed by all, under the influence of moderate cold or of excitement. But a contractile tissue, *not* muscular, may, we should conceive, give rise to it.

2. *Representative of the Omental Process of the Testis of the Rodents.*—"A small body of an irregular shape and variable size, and of a pale red or pinkish hue, is commonly found attached either to the upper extremity of the testis, or at the angle where the tunica vaginalis passes from the body of the gland to the epididymis. It is composed of a duplicature of this membrane, containing some fine cellular tissue and a number of small vessels. I have seen this little body in the testis of the fœtus whilst in the abdomen; and in early life it is often of proportionally larger size, and of a deeper red colour than in the adult. It is quite distinct from the pedunculated cysts often found attached to the head of the epididymis. This little appendage to the tunica vaginalis seems to correspond with, and to be a type of, the remarkable omental process attached to the superior part of the testis in the *Rodentia* and other animals. That it is an unimportant structure in the adult is shown by its being frequently wanting."

3. *Do the Ligamentous Columns of the Tunica Albuginea exist?*—Sir Astley Cooper, in his account of the Tunica Albuginea Testis, represents the cavity of the tunica albuginea as traversed by numerous ligamentous cords, which pass from the mediastinum or rete testis, across to the anterior edge of the organ, supporting and packing the lobes. On this Mr. Curling observes:—"I have not been able to make out any such ligamentous processes, passing into the substance of the testis, as are represented in Sir A. Cooper's work (part i, pl. 2. fig. 3), which I have found to be an exaggerated view of the preparation from which it was taken. The cords described appear to me to consist chiefly of blood-vessels supported by slight fibrous processes from the tunica albuginea and cellular tissue. In a well injected testis very little tissue of the nature of ligament can be found between the lobes." So far as our own dissections have gone, they quite coincide with those of Mr. Curling.

4. *The Tubuli Seminiferi.*—The lobes of the testis, which are estimated by Krause at 404 to 484, are not entirely distinct, but communicate with one another by means of anastomoses between the tubuli. These anastomoses increase in frequency towards the circumference of the testicle. "The tubuli thus form one vast network of communication, so that it is impossible to isolate completely either a duct or a lobule. The credit of making this interesting discovery of the anastomoses of the seminal tubes is due to Lauth. In only one instance did he succeed in finding a duct terminating in a blind pouch, and this he regarded as exceptional. Blind ends have been found, however, more frequently by Krause. The anastomoses of the tubules have been observed in the rat and other animals as well as in man."

The following are the approved measurements, &c. of the tubuli.

"The tubuli are of a white colour and uniform size, but their calibre differs in different subjects, and varies a good deal according to the age of the subject and the state of activity of the testes, being larger in young adults and when distended with semen than in old persons and when the gland is in a state of rest. The size of the ducts also often differs in the two testes of the same subject. In general the calibre of the tubuli corresponds to the size of the testis. Observers do not exactly agree in their estimates of the diameter of the tubuli. The average diameter of the uninjected canal is estimated by Muller at $\frac{1}{18}$ of a line, by Lauth at $\frac{1}{185}$ of an inch. Krause found the tubuli, when filled with semen, to measure about $\frac{1}{12}$ of a line, and in old men and youths $\frac{1}{16}$. Monro reckoned the number of the seminiferous tubes at 300; Lauth made the average number 840, and he estimated the mean length of all the ducts united at 1750 feet. He found the individual ducts to vary in length, the mean being 25 inches. Krause estimated their entire length at 1015 feet. The membrane composing the tubuli is of a mucous character, as has been clearly proved by microscopic examination, and it is continuous with the mucous surface of the genito-urinary system. There is no appearance of an intertubular substance; the ducts are merely connected by a loose network of vessels, and consequently readily admit of being separated and unravelled." 18.

5. *Length of the Epididymis.*—Possibly some of our readers may suppose that it signifies not much, whether the canal of the epididymis is a little more or less in diameter, or some inches plus or minus in length. But anatomists are exact people and deal in precise admeasurements. The only pity is, that they can't agree. For example:—

"The body and tail of the epididymis are entirely made up of the convolutions of the single canal in which the vasa efferentia terminate, closely connected by cellular tissue. Monro described this canal as gradually increasing in size from the head to the tail, and he estimated its calibre about its middle at $\frac{1}{80}$ of an inch. Lauth states that its size is subject to great irregularities in different parts and in different subjects. This anatomist has particularly described the convolutions of this duct, and has shown that they are regularly arranged in four series, which successively increase in size, the first being the smallest, and the fourth the largest." "Monro estimated the length of the canal at thirty feet eleven inches. Lauth found its mean length to be nineteen feet four inches eight lines. The parietes of the canal are strong and bear considerable resistance. The canal of the epididymis terminates in the excretory duct of the testis, the vas deferens, and is usually contracted at the part where the two join. It was calculated by Monro that the semen before arriving at the vas deferens traverses a tube forty-two feet in length. Lauth, however, makes the whole distance but little more than twenty-two feet." 23.

The slight difference between the measurement of these respectable anatomists is no more than as 2 to 1, which, considering that the question is a foot-rule one, must be looked upon as very moderate, and calculated to inspire great confidence in researches of this description.

6. *Muscularity of the Vas Deferens.*—This is unequivocal in the bear, bull, and other animals, but Mr. Curling has not been able to discover, with the microscope, more than simple fibrous tissue in the human vas deferens.

7. *The Spermatic Veins have Valves.*—Though many anatomists say no, Mr. Curling has observed valves in the larger veins. But they are seldom seen very near the testis, nor in the smaller veins, nor within the abdomen.

These are the only points that we can notice in Mr. Curling's account of the anatomy of the testis. We turn now to his description of—

*The Testis in the Fœtus, and its Descent into the Scrotum.*—The following is his description of the Gubernaculum.

" Attached to each testis whilst in the abdomen is a peculiar body which was termed by Mr. Hunter, who first described it, the *gubernaculum.* It is a soft solid projecting body of a conical form, which varies somewhat in shape and size at different periods of the testicular descent, becoming shorter and thicker as the gland approaches the abdominal ring. It is situated in front of the psoas muscle, to which it is connected by a reflexion of peritoneum. Its upper part is attached to the inferior extremity of the testis, lower end of the epididymis, and commencement of the vas deferens. The lower part of this process passes out of the abdomen at the abdominal ring, and diminishing in substance and spreading, terminates in three processes, each of which has a distinct attachment. The central part and bulk of the gubernaculum is composed of a soft, transparent, gelatinous substance, which, on examination in the microscope, is found to consist of nucleated cells, the primitive cellular tissue: this central mass is surrounded by a layer of well-developed muscular fibres, which may be distinguished by the naked eye, and which can be very distinctly recognised in the microscope to be composed of 'striped elementary fibres.' These muscular fibres, which may be traced the whole way from the ring to the testis, are surrounded by a layer of the soft elements of the cellular tissue similar to that composing the central mass; and in the same way as the testis the whole process, except at its posterior part, is invested with peritoneum. On carefully laying open the inguinal canal, and gently drawing up the gubernaculum, the muscular fibres may be traced to the three processes, which are attached as follows: the external and broadest is connected to Poupart's ligament in the inguinal canal; the middle forms a lengthened band, which escapes at the external abdominal ring, and descends to the bottom of the scrotum, where it joins the dartos; the internal passes in the direction inwards, and has a firm attachment to the os pubis and sheath of the rectus muscle. Besides these, a number of muscular fibres are reflected from the internal oblique on the front of the gubernaculum. It thus appears that the attachments of the muscle of the gubernaculum and those of the cremaster in the adult are exactly similar. I have succeeded in tracing out the former before the testis has descended, at different stages of the process, and immediately after its completion; and of the identity of the two no doubt can be entertained. Carus was of opinion that the cremaster does not exist before the descent of the testis; but that it is formed mechanically, by the testis pushing before it the lower fibres of the internal oblique, so as to form the loups of this muscle. This view which has been adopted by M. Jules Cloquet, and after him by many of the anatomists of this country, is, as I have shown elsewhere, clearly erroneous and inaccurate." 31.

We have made some dissections of the Gubernaculum, but we cannot boast of having perceived its muscularity with "the naked eye," so readily as Mr. Curling appears to have done. Nor can we avoid thinking that he has been felicitous in his dissections, making out, as he does, not only muscular fibres, but an elaborate series of origins and insertions.

It seems to need confirmation, *that* the Gubernaculum, which, if muscular, must have for its object to drag the testis down, should be transformed into the cremaster, whose object is to pull it up—*that* the scrotal fibres should, in the first place, in opposition to the common law of muscular contraction, contract *throughout their whole length,* reduce themselves, as it were, into nothing, and disappear, while the cremasteric fibres, so subordinate at first, should supersede the scrotal, and become largely developed.

Much as Mr. Curling condemns M. Jules Cloquet's account of the cremaster, we confess that it seems to us to tally more with actual and indisputable dissection, with what is obvious and demonstrable, than does Mr. Curling's. He owns that "a number of muscular fibres are reflected* from the internal oblique on the front of the gubernaculum." Those who doubt the correctness of his views may suspect that his ascending fibres of the gubernaculum are not altogether free of connexion with that internal oblique. The descending fibres of the gubernaculum, on the other hand, may be considered *the* gubernaculum, muscular in Mr. Curling's eye, not so in that of others.

In making these remarks, we would not be understood to deny the justice of our author's observations. They *may* be perfectly correct. But we cannot say that they are so demonstrably proved, nor so intrinsically probable, as to be beyond the reach of question, nor, without corroborative dissections by others, can the nature of the gubernaculum be fairly looked on as decided. It is right, however, to let Mr. Curling complete his demonstration and explain his own view fully.

"In the passage of the testis from the abdomen to the bottom of the scrotum, the gubernaculum, including its peritoneal investment and muscular fibres, undergoes the same change as that which takes place in certain of the *rodentia* at the access of the season of sexual excitement; the muscle of the testis is gradually everted, until, when the transition is completed, it forms a muscular envelope external to the process of peritoneum, which surrounds the gland and front of the cord. As the testis approaches the bottom of the scrotum, the guber-

---

* *Reflected.* This term is strangely abused by some anatomists. Reflected means turned or bent back. How can the fibres of the internal oblique be so contorted. They maintain their natural direction, and pass across the front of the gubernaculum.—*Rev.*

naculum diminishes in size, owing to a change in the disposition of its cellular elements: the muscular fibres, however, undergo little or no diminution, and are very distinct around the tunica vaginalis in the recently-descended testis. The mass composing the central part of the gubernaculum, which is so soft, lax, and yielding, as in every way to facilitate these changes, becomes gradually diffused, and after the arrival of the testis in the scrotum contributes to form the loose cellular tissue which afterwards exists so abundantly in this part: the middle attachment of the gubernaculum, which may be traced to the dartos at the bottom of the scrotum, gradually wastes away, and soon becomes indistinct, though slight traces of this process often remain to the latest period of life. Thus, after death, in dragging the testis of an adult out of the scrotum by pulling the cord, the lower part of the gland, which is uncovered by serous membrane, is often found connected to the bottom of the scrotum by a band of firm and dense cellular tissue, which requires division with the scalpel. This band is the remains of the middle attachment of the gubernaculum. In cases in which the testis has been retained in the groin, I have traced a cord of dense tissue from the gland to the lower part of the scrotum. After the arrival of the testis in the scrotum, the peritoneum with which it is closely invested, its original envelope, becomes the inner layer of the tunica vaginalis; whilst the pouch around, which is continuous with it, forms the outer layer, or vaginal sac. Immediately after the descent of the testis, this bag communicates with the abdomen, and in quadrupeds continues to do so during life; but in the human subject it soon begins to close, and when the fœtus is ushered into the world, the abdominal orifice is often shut, and the whole canal from the ring to the upper part of the testis is in general completely obliterated in the course of the first month after birth. The obliteration is effected by an intimate union of the surfaces of the serous membrane. It sometimes does not take place at all, or is delayed or only partially completed. Congenital hernia, or hydrocele, is the result of a failure in this process; and other forms of hydrocele are occasioned by imperfect obliteration of the canal, as will be hereafter explained.

“ Much difference of opinion exists as to the immediate cause of the descent of the testis. Hunter, Meckel, and others, came to the conclusion that the muscular fibres of the cremaster are insufficient to bring the testis lower down than the abdominal ring, and complete the descent. They were not, however, acquainted with the attachment of this muscle to the pubes external to the ring, or it would be difficult to understand why Mr. Hunter, after arriving at the conviction that the cremaster passes up to the testis whilst in the abdomen chiefly from analogy, was not induced by the same process of reasoning to conclude, that a muscle capable of drawing down the testicle in animals would be adequate to accomplish the same purpose in the fœtus. The necessity for some active agent to effect this change in the latter would appear to be greater even than in animals, since, in the usual position of the fœtus in utero, the passage of the testis is contrary to gravitation, and unaided by the movements of respiration. Now, when we consider the attachments and connexions of this muscle in the fœtus, the perfect condition of its fibres as ascertained by microscopical examination, and the circumstance that there are no other means, no other motive powers by which this change can be effected, or in any way promoted, I think there is no reason to doubt that the cremaster executes the same office in the human embryo, as that which it undoubtedly performs in certain animals at a particular season. The fibres proceeding from Poupart's ligament and the obliquus internus tend to guide the gland into the inguinal canal, those attached to the os pubis to draw it below the abdominal ring, and the process descending to the scrotum to direct it to its final destination. As the descent approaches completion, the muscular fibres which perform so important a part in it gradually become everted, and, instead of drawing down the testicle, acquire the new functions of elevating, supporting, and compressing it.” 36.

We have a full account of the *Functions of the Testis*. They are tolerably well known. Mr. Curling has several times discovered spermatozoa in the testes of men upwards of seventy years of age, and once in the testicle of a tailor who died at the age of eighty-seven; and there are instances on record of persons retaining the procreative faculty to the age of one hundred years. The tailor certainly did not bear out the proverb so derogatory to the manhood of his craft! Old Parr, who lived to the great age of 152, was found by Harvey to rejoice in testiculis *integris et magnis*. Mr. Curling gives some sound moral advice, which may be recommended to young men.

Before we quit this account of the Anatomy of the Testis, we must say that there does not appear in it a sufficient amount of interest or novelty, to have rendered its introduction into the volume necessary. The few original observations of the author might have formed the subject of a paper elsewhere, or might have been incorporated in a brief introductory chapter. The fault, however, if it be one, is of no great moment, as the reader, if he likes, may *skip* what the writer, we dare say, took a very great deal of pains with.

We pass to the Diseases of the Testis.

### I. CONGENITAL IMPERFECTIONS AND MALFORMATIONS.

The first section of this first chapter is occupied with *Numerical Excesses and Defects*.

An account is first given of *Supernumerary Testes*. Nature has not been liberal in this respect; for most of the instances on record are too apocryphal or vague to be relied on. Perhaps as good an instance as any is the following:—

"Dr. Macann, staff-surgeon, relates, that on examining a recruit about twenty years of age, a body was found on the right side of the scrotum, which was so similar to the two testicles in size, form, feeling, and consistence, as to leave no doubt of its being a third testicle. This body was situated between the groin and the proper testicle of the right side, with which, however, it did not seem to be in immediate contact, but to be suspended, as it were, by a shorter cord or hung up in a separate sac. The right spermatic cord was much thicker than natural at its upper part, where, in fact, it consisted of two cords, one of which was distinctly traced into the upper testicle on this side, and the other, much longer, into the lower testicle, and in each of those parts as well as in the cord on the left side, the vas deferens could be distinctly felt, like a piece of whipcord, between the fingers. The man stated that the third testicle had occupied its present situation as long as he could remember, and had never caused him any inconvenience." 51.

A piece of omentum, a fatty or fibrous tumor in the scrotum, or an encysted hydrocele of the cord, might be readily mistaken for an extra testis. Dr. Monsey thought he was favoured, but he had only an indurated fibrous tumor attached apparently to the tunica vaginalis.

The converse of too many testes is, of course, *too few testicles*. There have been many reported instances of *monorchides* or one-testicled men: most of them were, probably, cases in which one remained in the abdomen, or had been completely atrophied. Mr. Curling quotes a case from Mr. Paget, which is not quite conclusive; and another from Dr. Fisher, of Boston, of a more satisfactory character. In a case of Mr. Thurnam's, one testis would appear not to have been formed, the other not to have descended.

A curious instance of *union of the two testes* in the abdomen, along with the two kidneys and suprărenal capsules is detailed.

*Deficiencies and Imperfections of the Vas Deferens.*—These are considered rather in detail. Several instances are quoted in which the vas deferens, of one or both sides, terminated in a cul-de-sac, or in an imperfect vesicula seminalis. The duct, too, has been found wanting throughout nearly its whole extent.

" Mr. Paget has happily explained the origin of these several defects in the vas deferens by reference to the mode of development of the special organs of generation. He observes, after Müller and Valentin, that, in the normal course of human development, the proper genital organs are in either sex developed in two distinct pieces: namely, the part for the formation of the generative substance, the testicle or ovary, and the part for the conveyance of that substance out of the body, the seminal or oviduct. The testicle or ovary, as the case may be (and in their earliest periods they cannot be distinguished), is formed on the inner concave side of the corpus Wolffianum; and the seminal or oviduct, which is originally an isolated tube closed at both extremities, passes along the outer border of that body, from the level of the formative organ above to the cloaca or common sinus of the urinary, genital, and digestive systems below. The perfection of development is attained only by the conducting tube acquiring its just connexions at once with the formative organ, and, through the medium of the cloaca, with the exterior of the body. The sexual character is first established, when, in the male, the formative and conducting organs become connected by the development of intermediate tubes which constitute the epididymis; or when, in the female, a simple aperture is formed at the upper extremity of the conducting tube, and is placed closely adjacent to the formative organ. In both sexes alike, the lower extremities of the conducting tubes first open into the common cloaca, and subsequently, when that cavity is partitioned into bladder and rectum, or bladder, vagina, and rectum, they acquire in each their just connexions, and become in the male the perfect vasa deferentia, and in the female the Fallopian tubes and uterus.

" Now in Brugnone's case, and in Bosscha's, we have examples of one of the male conducting tubes being developed in only a very small portion of its natural extent. These, therefore, clearly confirm the description just given; for they prove that the testes may be formed quite independently of the vasa deferentia. In the other cases the vas deferens was probably formed originally in its whole length; but it seems to have failed of acquiring its due connexion in the one series of defects at the end next to the testicle, and in the other at the end next to the bladder." 62.

Such views are ingenious, and probably not very wide of truth. But they must not always be implicitly received. Nothing could be more simple, or, seemingly, more satisfactory, than the explanation of hare-lip, cleft palate, &c., by the occurrence of arrest of development in the mesial line, and imperfect fusion of the lateral halves of the organs. Yet M. Cruveilhier assures us that at no period of fœtal development has he seen such halves or their coalescence. Facts are yet to be rigorously observed before the generalizations of transcendental anatomy can be implicitly received.

There is a more practical question in the back-ground—what influence have deficiencies and imperfections of the vas deferens on the evolution of the testis? Let Mr. Curling reply :—

"In the case of the adult which occurred at St. Bartholomew's Hospital the testis was small, and its structure appeared granular, like the undeveloped testis of a youth; but as it had not descended into the scrotum, and was combined with hernia, there may have been other causes impeding its due evolution. In Mr. Hunter's case, the testicles which were in the scrotum were very sound. In the case of the man related by Brugnone the testis on the side corresponding to the defective vas deferens was perfectly sound, and nearly of the same size as the other. So also in Bosscha's case it is stated that the testis was sound. Although either of these defects in the vas deferens renders the gland an useless organ, and if it occurred on both sides of the body would necessarily cause impotency, these cases, nevertheless, tend to show that the absence or imperfection of the excretory duct does not prevent the development of the testis at the proper period, and has no direct influence in causing it to waste, and these inferences are fully confirmed by experiments on animals." 63.

Experiments of Sir Astley Cooper's and of our author's own, are appealed to in confirmation of this view.

Some years ago, it was proposed to cure certain diseases of the testis, by destroying a portion of the vas deferens, it being contended that wasting of the gland would be the consequence. We argued, at the time, that this was proceeding on a false analogy, and that atrophy of the organ would probably not follow. The statements found above confirm this opinion.

*Imperfect Descent of the Testis.*

One or both testes may, as is well known, be detained, at birth, in the abdomen, in the inguinal canal, or in the groin just outside the external abdominal ring. In a table of one hundred and three male infants examined by Wrisberg at the time of birth, it appears that seventy-three had both testes in the scrotum; in twenty-one, one or both were in the groin; of these, five had both, seven the right, and nine the left in the groin; in twelve, four had both, three the right, five the left, only in the abdomen. It would almost appear that, in the majority of instances, the imperfection is most frequent on the left side. This, however, is scarcely borne out by the observations of others.

Sometimes the testis continues fixed in the improper situation. Mr. Curling is of opinion that unless it descends within a twelvemonth after birth, it is rarely completed without an accompanying hernia. When the descent is not completed before puberty, uneasiness in the part is sometimes experienced, from the enlarging gland being retained in the inguinal canal.

The following are Mr. C.'s ideas on the cause of imperfect descent of the testis :—

"It is clear that there must not only be a perfect adaptation of parts, a due relation between the body drawn down, and the structures which it traverses, but also corresponding power in the agent by which it is accomplished. There are few muscles in the human body whose development in different individuals varies in a greater degree than that of the cremaster. And if such be the case after birth, it is not unreasonable to presume that similar differences exist in the fœtus before the descent of the gland, and that a failure in that process may be the result of deficient power in the musculus testis to accomplish the passage. May we not also conclude that this muscle is sometimes paralysed, and that the faulty descent is owing to a want of a due supply of the nervous energy which we know is often denied to other muscles during fœtal existence, and is the cause of deformities in the feet and other parts with which infants are often ushered into the world? I think, indeed, we may fairly enumerate paralysis and defective development of the cremaster amongst the causes of the imperfect descent of the testis." 69.

Sometimes adhesions, the consequence of intra-uterine peritonitis, connect the testis to the viscera, &c. in the abdomen. Many instances of this are cited. Mr. Hunter was of opinion that the obstruction was often at the outer ring, and the frequent persistence of the testis in the groin seems to give a colour to the notion. In short, Mr. Curling concludes that the failure in the descent of the testis may result from want of power or paralysis of the cremaster muscle, from adhesions retaining the gland within the abdomen, and from a contracted state of the opening of the external abdominal ring.

*The Condition of the Undescended Testis* is next touched upon by Mr. Curling. It is well known that Mr. Hunter believed that a testis which remains in the abdomen is imperfect, and remains *because* it is so. The only case that he saw and has described contradicts his opinion. What anatomical evidence exists upon the subject leans in the same direction. We conceive that if a person whose testes have not quitted the abdomen presents the appearances, feels the passions, and displays the ordinary evidences of the powers of a man, he has no great reason to dread incompetency or accuse his fate. But if his appearance is unmanly, his passions weak, and their manifestation imperfect, the affair has a different aspect. The proof of the pudding, in fact, is in the eating, and the virility of any given individual is only to be settled by experience. We are not warranted in predicting that a child whose testes do not descend will necessarily prove sexually incompetent as an adult, for the evidence, so far as it goes, and the presumption, are, on the whole, in his favour.

*When arrested in the inguinal canal,* the testis would appear to be more frequently atrophied than when retained in the abdomen. In the former case, "the organ is liable to be compressed during any violent action of the abdominal muscles, and even in acute flexion of the thigh, as in walking up stairs, and on bending the body forwards whilst in the sitting posture. It is exposed to injury from blows which, being fixed it is unable to elude, and to pressure from the frequent manipulation of the surgeon, and the ruder handling of bandage-makers, and often through ignorance from the application of a truss. It occasionally happens that a testis, after retention in the abdomen, without any uneasiness having been experienced, passes into the inguinal canal, and sometimes appears at the external ring, playing backwards from one situation to another. When this is the case, the gland is liable to be caught at some particular part by a sudden contraction or spasm of the abdominal muscles, which gives rise to violent pain and suffering, and a sickening sensation which lasts for some hours unless relieved by the hot bath, fomentations, and opiates." Mr. Curling, therefore, thinks a testis in the abdomen "in a more satisfactory state," than one in the groin.

"On this account, and as the descent is seldom perfectly accomplished when delayed beyond the age of ten or twelve, I think it becomes a serious consideration in cases where the gland does not make its appearance till this late period, whether the well-being of the patient would not be best consulted by our employing some mechanical means to prevent the escape of the organ from the abdomen. A strong reason for adopting this practice is afforded by the great liability to rupture which exists in all cases of the tardy descent of this organ, owing to the persistence of a sac ready prepared for the reception of a protrusion, and in many instances to adhesions between the testis and intestine or omentum. A hernia may occur whilst the testis is still in the abdomen, or after it has passed the ring, and the viscera may descend into the scrotum, the gland being detained in the groin. Cases of this kind are very embarrassing, as it is seldom practicable to fulfil the two opposite indications of preventing the protrusion of the viscera, and encouraging the descent of the testis." 80.

We are inclined to agree with Mr. Curling in this recommendation. We certainly conceive that a testis in the abdomen is in a better situation than one in the groin, and cannot perceive the advantage of aggravating a hernia for the sake of obtaining what is only an imaginary good, perhaps a real evil.

Mankind will not always reason philosophically, and the prejudice is unquestionably in favour of visible and tangible testicles. A medical student, in whom they had not descended, committed suicide in consequence. He ought to have *known* better, the more so, as when the body was examined the testes in the abdomen were nearly, if not quite, their natural size, and the ducts contained semen.

"It occasionally happens that a testis descends into the upper part of the scrotum accompanied with a reducible hernia without adhesion to the gland. In such a case the rupture may be reduced without the testis, and admit of the application of a truss, which serves the double purpose of preventing the hernial protrusion and preventing the testis from reascending. Cases, however, in which this practice can be adopted are very rare, for frequently the rupture cannot be returned without the testis, and, in many cases, it is impossible to apply the truss without painful pressure being made upon the gland." 83.

An operation was attempted in one case, at Munich, with the view of bringing a testis from the groin to the scrotum and fastening it in that situation. The success seems to have been problematical, and the experiment altogether one of dubious propriety.

Mr. Curling dwells upon one disadvantage attending the imperfect descent of the testis, more particularly when it remains in the abdomen—we allude to the more serious consequences which result from its diseases. Instead of being connected with a small serous membrane, the tunica vaginalis, the organ is then in contact with the peritoneum itself, and extension of inflammatory action to this is a matter of no slight moment. The following cases are detailed by Mr. Curling as instances of this description.

*Case.*—"A lad, ten years of age, was admitted into the London Hospital from a distance in the country, dangerously ill. His mother, who came with him, stated that on returning from school four days before, he was kicked in the right groin by one of his school-fellows. He suffered great pain at the time, and on the following day became very ill. Having continued to get worse, he was brought to the hospital. The boy was evidently seriously ill from the effects of acute peritonitis. He was almost in a state of collapse; his countenance was anxious; his pulse quick, small, and feeble; his abdomen hot, tumid, and extremely tender; and his bowels constipated, but they had been opened since the accident. There was a considerable diffused swelling in the right groin, and the right side of the scrotum was empty. The boy's state was such, that no active means could be taken to relieve him, and he died in twelve hours after his arrival at the hospital. On examination of the body, marks of extensive peritonitis were found throughout the whole of the abdominal cavity, the viscera being coated with lymph, and a turbid serum abundantly effused. In the right iliac fossa, just beneath the peritoneum, were seen two small abscesses of recent formation. An atrophied testis was discovered close to the external ring, amongst a mass of cellular tissue, infiltrated with pus and lymph. There were indistinct traces of a tunica vaginalis continuous with the peritoneum. I apprehend that, in this case, the blow occasioned inflammation in the testis and surrounding parts, which, extending to the peritoneum, caused the patient's death." 86.

This case does not appear to us to be very conclusive on the point. The testis was atrophied and is not shewn to have contributed in any way to the inflammation, while the "indistinct traces of a tunica vaginalis continuous with the peritoneum" can scarcely be allowed to go for much. It is far from improbable, that the injury gave rise to inflammation and suppuration in the cellular membrane of the inguinal canal, and that this extended directly and continuously to the peritoneum. Be this as it may, the case is interesting.

*Case* 2.—" I was summoned one evening to the hospital to see a patient of Mr. Luke's, who was supposed to have strangulated hernia. On my arrival I found the patient, a stout labourer, aged 33, and a married man, with a considerable swelling in the right groin, which was of an oval form, received a slight impulse on coughing, and was more solid and tender than is usually the case with a rupture. The house pupils had made unsuccessful attempts to reduce the swelling, which gave the man much pain. He stated that he was subject to a swelling in the groin, which occasionally came down in the daytime and disappeared at night, but he had never worn a truss. It descended the evening before, and caused considerable pain; and although it went away during the night, the abdomen had continued painful during the day. Whilst straining himself at work in the evening it again made its appearance; and as it occasioned considerable pain, he came to the hospital for relief. The abdomen was tender on pressure and he complained of pain in it chiefly in the vicinity of the umbilicus. He did not feel sick, and his bowels had been open twice during the day. The pulse was full and hard. There was no testicle on the right side of the scrotum, but the left was in its natural situation, and of proper size. I concluded that the tumour consisted of a retained testis which had been accidentally protruded at the external abdominal ring, and become inflamed from pressure, and that the inflammation had extended to the peritoneum, the latter membrane being, however, only slightly affected. I could not quite satisfy myself whether a portion of intestine had accompanied the testis, though this appeared very probable. I ordered the man to be bled to ℥xvj., fourteen leeches to be applied over the swelling, and a brisk cathartic to be given him. He continued in suffering during the early part of the night, but having dropped asleep he found on awaking that the swelling had disappeared. The bowels were relieved in the course of the morning, but the groin and abdomen continued tender for two or three days. There was still a tendency to reprotrusion of the testis and intestine when the man coughed. A truss therefore was applied as soon as the pressure of it could be borne, which was six days after his admission, when he was discharged." 87.

Here was certainly inflammation of an undescended testis, but the symptoms can scarcely be said to have been violent, not so much so indeed as we have seen them in common "hernia humoralis." It is possible, however to conceive such a case pushed to a dangerous degree, and it is probable that if the non-descent of the testis were an ordinary or a very frequent occurrence, dangerous inflammation of the peritoneum might now and then be the consequence.

Mr. Curling makes some judicious remarks on the *Diagnosis in Cases of Imperfect Descent of the Testis.* They may be loosely said to resolve themselves into this—always look if the testis is in the scrotum. If it is not, a case of suspicion lies against the tumor in the groin, and the surgeon must exercise his judgment and sagacity in forming an opinion on its nature.

*Descent of the Testis into the Perinæum.*—It is difficult to say why this should occur, or how it can happen, unless there be some deviation from the normal condition of the parts. Mr. Hunter saw one case, and was consulted on another. A case, too, occurred at the London Hospital. The testis is exposed to injury in sitting, and, of course, in riding on horseback. Mr. Hunter advised that the organ should be supported in a situation near the groin, by the application of a bandage that might hinder its descent into the perineum, by which the parts might be in time so consolidated as to retain it by the side of the scrotum.

### ATROPHY OF THE TESTIS.

Mr. Curling considers this under two heads—1, Arrest of Development; and 2, Wasting.

*Arrest of Development.*—There are few members of the profession who have not witnessed cases, more or less marked, of this. An instance related by Mr. Wilson is curious, from the sudden start which the organs made. Mr. W. was consulted "by a gentleman twenty-six years of age on the propriety of entering the marriage state, whose penis and testicles very little exceeded in size those of a youth of eight years of age. He had never felt the desire for sexual intercourse until he became acquainted with his intended wife; since that period he had experienced repeated erections, attended with nocturnal emissions. He married, became the father of a family; and these parts, which at six and twenty years of age were so much smaller than usual, at twenty-eight had increased nearly to the usual size of those of an adult man."

In reference to this subject it is interesting to notice the connexion between imperfect development of the testes and defective organization of the brain. Mr. C. refers to two cases of idiots, in which the testes were very imperfectly developed. Mr. C. adds:—

"I have remarked that this gland, when retained in the abdomen or inguinal canal, does not in general acquire its complete state of development, and that, though frequently capable of secreting, it is commonly small in size. I have also noticed, in cases of congenital inguinal hernia, that the testis, even in its natural situation, was not of its proper size at the period of puberty; so that when the infirmity existed on one side only, the testis was not more than half or two-thirds the size of the other gland. The arrest of growth in this latter case is attributable to the combined effects of the pressure of the protruded intestine on the vessels of the cord, and to the obstruction to the circulation caused by the application of trusses and bandages to the groin." 98.

### Wasting of the Testis.

Mr. Curling observes that, to determine what is or is not wasting of the testis, we must first fix something like a standard weight of the organ. From numerous experiments, the weight was found, in healthy adults, to vary from four drachms to nine, the mean weight being six drachms. In the most lingering cases of phthisis and in other emaciating diseases the organ was never found to weigh less than three drachms. An adult testis weighing less than three drachms he would consider atrophied.

"A testis in an advanced state of wasting, not arising from disease of the gland, usually preserves its shape, but feels soft, having lost its elasticity and firmness. Its texture is pale and exhibits few blood vessels, tubuli and septa dividing the lobes are indistinct, and the former cannot be so readily drawn out into shreds as before. The epididymis does not usually waste so soon nor in the same degree as the body of the testis. It sometimes, however, loses it characteristic appearance, and I have even found it reduced to a few fibrous threads. The fluid pressed out of the wasted testis and epididymis is entirely destitute of spermatic granules and spermatozoa. In many instances, adipose tissue is deposited behind the tunica vaginalis, and encroaches on the epididymis and posterior part of the testis. Mr. Gulliver has recently discovered fatty matter in the glandular substance of atrophied testes, and I have since had an opportunity of confirming this interesting observation." 99.

Mr. C. relates a case in point. He then goes on to say:—

"The structures composing the spermatic cord undergo a corresponding diminution; the cremaster muscle disappears, the nerves shrink, and the vessels are reduced in size and number. The vas deferens, though small, can generally be injected with mercury as far as the commencement of the epididymis. A testis atrophied from disease is not only of diminished size and weight, but is altered in shape, being uneven and irregular and sometimes of an elongated form. The surfaces of the tunica vaginalis are adherent, and its cavity is partly or entirely obliterated. There is no, or very little, trace of the proper glandular structure, the organ being converted into fibrous tissue of a firm texture. It loses its peculiar sensibility to pressure, but is sometimes the seat of morbid sensibility." 100.

Impeded circulation, pressure, want of exercise, loss of nervous influence, may be naturally supposed to operate as causes of atrophy of the testicle. So an aneurysm of the aorta, at the origin of the spermatic arteries, the application of a truss, the pressure of dilated veins, ligature of the spermatic artery have given rise to the affection.

Mr. Curling inquires if chastity has this effect. He thinks not. It is probable, however, that the testis offers no exception to the general physiological law—that the moderate exercise of an organ contributes to its development and perfection, its inaction to the reverse. The following case seems to support such an opinion.

*Case.*—" A man fifty-two years of age, and much emaciated, was brought to the hospital in a half-insensible state, and sinking, in the severe winter of February, 1838. There was an extensive ulcerated sore in the perineum, through which the urine dribbled away, no water passing by the penis. The man could give no account of himself; but his wife stated that he underwent an operation in the perineum, at the hospital, about twelve years ago; that the water had passed from underneath ever since, and that he had long been out of health. He gradually sank, and died on the third day after his admission. On examination of the body both lungs were found hepatised, but the heart and abdominal viscera were sound. Both kidneys were diseased. The bladder was contracted and empty. The prostate was converted into a multilocular purulent cavity, the urethra covering it being riddled with the enlarged prostatic openings. Two ulcerated apertures, each a quarter of an inch in diameter, in the membranous part of the urethra, communicated with the sore in the perineum. The urethra was completely obliterated and impervious near the meatus, and strictured in other parts. Both testes were atrophied, being scarcely larger than hazel nuts, but the tubular structure was still apparent. There was a small hydrocele connected with the right testis. The severe disease of the urinary organs must have incapacitated this man for sexual connection for a long period of years, and to this circumstance we must chiefly attribute the atrophy of the testes, since the organs were not otherwise diseased." 104.

Mr. Curling has never seen atrophy of the testis from enlargement of the prostate. It sometimes happens in paraplegia, and from injury of the spine. The most common cause, he thinks, is inflammation. Mumps, by metastasis, give rise to it. Abuse of venery and onanism lead to it—not very unfrequently if we may trust to our own observation. Mr. C. doubts the reputed effects of iodine—so, we must admit, do we. It follows injuries of the head.

*Case.*—" A few years ago a man who had met with an injury of this description, which had been followed by wasting of the testes, and the development of tumours on each side of the chest, resembling mammæ, presented himself at the different hospitals in London. I saw him in March, 1828, at the London Hospital, when he had the appearance of a man who had seen hard service. He stated that he was about fifty-nine years of age, a married man, and the father of several children. He had belonged to the legion in the Queen of Spain's service. About two years and a half previously, in an attempt to jump over a trench in a retreat, he fell backwards, and injured the posterior part of his head. Whilst on the ground he received a bayonet wound on the left side, and a sabre cut on the forehead of the same side. He recovered from these injuries, and returned to England. Since the accident he had completely lost his virility. He had no desire for sexual connection; his penis had dwindled in size; his right testis had gradually wasted, and was no larger than a horse bean, and the left gland was also a good deal diminished in bulk. The skull at the occiput seemed somewhat flattened." 108.

Some cases of a similar description are quoted from Larrey, Hennen, and Lallemand. Mr. Curling remarks on them :—

" We cannot doubt that in these cases the loss of sexual desire and the wasting of the testes were the direct results of the injury to the brain, and they go far to prove the essential dependence of the functions of these glands upon the cerebral organ. The physiologist cannot fail to notice the rapidity with which the atrophy is stated in some of the cases to have succeeded the injury, and the extent to which it proceeded. The withering of the testicles was, indeed, so remarkable, that it can only be attributed to the sudden and complete extinction of the sexual instinct resident in the brain, and (if I may so express myself) to the immediate impression on the system of the future uselessness of these organs. In old age and in lingering diseases the decay of the testicles is extremely slow and gradual, and is never carried to the extent observed in cases of injury to the brain. In fact, men have survived the power or desire of performing the sexual act many years without the testes being materially reduced in size. We have seen, too, that in animals the testes have been rendered useless by interrupting the vasa deferentia, without any such striking effect being produced on the glands as occurred in these cases of cerebral injury." 110.

It is à priori exceedingly improbable that an instinct so powerful as the sexual passion, and one that so influences the physical and moral constitution of the animal, should be unconnected in a direct and immediate manner with the cerebral centre. What reasoning would prompt us to presume, facts of the nature of those just related go far towards proving. Difficulties, no doubt, attend the subject, and the precise amount of influence exerted by the cerebrum or cerebellum on the generative organs, will probably never be determined.

Mr. Curling alludes to other instances of wasting of the testis, unaccounted for by any apparent cause. Thus, a well-grown boy between nine and ten years of age was observed to grow more delicate in his figure for some months. His mother discovered that his testicles had almost disappeared. On examination, it was difficult to find them, and when discovered, they did not seem larger than two full-grown peas. The mother asserted that he was born with them of the usual size, and that they continued to grow for some time afterwards. Baron Larrey relates, that in several soldiers of the army in Egypt, at the close of the campaign in 1799, the testicles almost entirely disappeared, for which he assigns no satisfactory cause.

The conclusion at which he arrives is far from encouraging. He observes:—

"An investigation of the causes of atrophy of the testis is sufficient to show that we have little power by any mode of treatment to promote the development or arrest the decay of this organ. They are commonly the result of actions beyond the surgeon's reach or control. In certain cases, as in atrophy from pressure, or an impeded circulation, we may, by judicious measures, assist in retarding the wasting process; but a statement of the circumstances which conduce to this change is sufficient to indicate the means required to check its progress." 111.

We cannot say that we agree altogether with Mr. Curling. Many cases of atrophy of the testis are unquestionably altogether beyond our reach, and in most the prospect is a gloomy one. But there are some, at all events, which are benefitted by treatment, and hold out some encouragement to the practical surgeon. We allude particularly to the atrophy which results from onanism or from excessive sexual indulgences. We have seen some remarkable instances of restoration of the organs, when the wasting had proceeded to such a degree as scarcely to justify hopes of recovery. Occasional connexion—the shower-bath—cold seabathing, &c.—country air and exercise—a generous diet—tonics—and proper attention to the secretions have seemed of essential service. We have thought that the combination of the tinctura ferri sesquichloridi with the tinctura lyttæ has been useful.

We pass over a Chapter on Injuries of the Testis, and one on Self-castration, which present nothing to detain us; unless, perhaps, we may pause to remark, that the testicle is more often wounded than some people suppose, in operations for hydrocele, and no great matter neither—and that cases of self-castration usually do well, severe as is the proceeding itself, without precautions respecting hæmorrhage, and with the disadvantage of that morbid state of mind which has usually prompted to the mutilation.

### HYDROCELE.

Mr. Curling first alludes to what has been termed *acute hydrocele*, inflamation of the tunica vaginalis. Its inflammatory changes resemble those of the other serous membranes. Adhesions are probably the usual result, but sometimes, as is well-known, the opposed surfaces do not unite although they cease to secrete. It is a curious fact that inflammation of the tunic is more apt to be propagated to the epididymis than to the body of the testis, which has been accounted for in this manner by Gendrin. "He says, when the subserous cellular tissue, which always participates in the inflammation of a serous membrane, penetrates into the interior of an organ, it becomes a ready means of communicating the inflammatory action; but when the contiguous organ or subjacent part is of a different structure from that of the cellular tissue, the extension of inflammation inwards is checked. Thus, in the case of the inflamed tunica vaginalis, the cellular tissue readily transmitted the morbid action to the epididymis, but the tunica albuginea arrested its progress to the body of the testis; and this explains the fact that, after inflammation of the tunica vaginalis excited by injection, the body of the gland is rarely found to suffer. On the other hand, the epididymis is seldom attacked with inflammation without the disease being quickly propagated to the tunica vaginalis."

Mr. C. presents a Tabular View, a good one, of the different Varieties of Hydrocele. We insert it. (*See next page.*)

We have first a good account of *Simple Hydrocele of the Testis.* Into the general history of so familiar a complaint it is unnecessary for us to enter. We shall merely select a point here and there for notice.

1. We have a sort of statistical view of the quantity of fluid contained in Hydroceles.

"In this country it seldom exceeds twenty ounces, though it has been known to amount to several pints. The largest quantity which I have met with is forty-eight ounces. Mr. Cline is said to have removed from Gibbon the historian as much as six quarts. From a table of 1000 cases of hydrocele which occurred at the native hospital of Calcutta, constructed by Dr. Dujat, it appears that the quantity of serum evacuated varied from less than ten to upwards of one hundred ounces. Of 370 cases of double hydrocele, the fluid was more abundant on the right side in 109, and on the left side in 128. Of the 630 cases of single hydrocele, in rather more than a third of the number the quantity of fluid was under ten ounces; in two sevenths it was from ten to nineteen ounces; in nearly a third from twenty to forty-nine; and in eighteen cases the quantity of serum was from 50 to 120 ounces." 127.

2. After noticing the form of *Hydrocele* termed multilocular, in which partial adhesions of the tunic produce a sacculated arrangement of it, the cysts sometimes not communicating with each other, Mr. C. goes on to say, that he has seen the sac divided into two distinct cavities, by a membranous partition (a circumstance which we also have witnessed), and he adds:—" There is one kind of sac or pouch often met with in hydroceles

---

- Hydrocele

  - Of the Testis

    - Simmple.

    - Congenital.

    - Encysted

      - Of the Epididymis.

      - Of the Tunica Albuginea.

      - Of the Tunica Vaginalis.

  - Of the Spermatic Cord

    - Diffused.

    - Encysted.

  - Complications of

    - Simple H. combined with Encysted H. of the Testis.

    - Simple H. combined with Encysted H. of the Cord.

    - Simple H. combined with Diffused H. of the Cord.

    - Oscheo-Hydrocele

      - Simple H. combined with Inguinal Hernia

      - Encysted H. of the Cord combined with Inguinal Hernia.

  - Of the Hernial Sac

    - True.

    - Spurious.

which does not appear to have been described. It is situated on the inner side of the testis; but the opening into it is always found on the outer side, between the body of the gland and the middle of the epididymis. This sac, which varies very much in size, is formed by the distention of the cul de sac which I have described as existing naturally at this part. Two examples of this kind of pouch in the Hunterian Collection were shown me by Mr. Paget."

3. *Age for Hydrocele.*—It would seem to prefer early infancy and middle age. "In sixty cases of hydrocele, M. Velpeau of Paris found,

| | | |
|---|---|---|
| Between the ages of | 15 and 20 ...... | 3 |
| | 20 — 30 ...... | 13 |
| | 30 — 40 ...... | 11 |
| | 40 — 50 ...... | 16 |
| | 50 — 60 ...... | 10 |
| | 60 — 70 ...... | 6 |
| | 70 — 80 ...... | 1 |

"In a table of 1000 cases of hydrocele treated by iodine injection at the Native Hospital of Calcutta, it appears that none of the patients operated on were less than eighteen years of age; about one twenty-fourth were not more than twenty years old; rather more than a sixteenth were from twenty-one to twenty-five years of age; a little less than half from twenty-eight to thirty-five; a little more than a quarter from thirty-six to forty-five; and an eighteenth were upwards of forty-six years."

Mr. Curling's remarks on the causes which lead to the frequency of Hydrocele are judicious. He observes:—"All circumstances which determine blood to the organ in excess, or impede its return to the heart, or which act in any way in disturbing the circulation through the gland, must be regarded as remote causes of the disease; and, considering the exposed and depending situation of the testicle, the liability of its vessels to obstruction, and the irregular nature of its functions, there can be no difficulty in accounting for the frequency of this affection. I shall hereafter have occasion to mention that hydrocele is often combined with inguinal hernia; a disease obviously very favourable to the effusion of serum in the tunica vaginalis, owing to the pressure of the rupture on the veins of the spermatic cord, and which is often increased by the use of trusses and bandages. Hydrocele is occasionally developed after a violent strain or great fatigue, or after a slight blow on the gland which was considered at the time to be too trivial to require attention. In many of these cases the effusion appears to originate in a low degree of inflammation of the tunica vaginalis. I have already stated that marks of previous inflammation are occasionally observed in the sacs of hydroceles. On examining the body of a man aged forty-nine, who died of apoplexy, I found about two ounces of serum in the vaginal sac of both testes, and also several old adhesions, and some spots of induration and thickening of the testicular portion of the membrane. I have observed similar appearances in other cases of incipient hydrocele, as well as imperfect multilocular cavities and septa, and induration, and enlargement of the epididymis, clearly evincing that the part had been the seat of inflammation. In some few instances I have met with hydrocele under circumstances which have led me to suspect that the disease was connected with, or sympathetic of, a chronic affection of the urethra, as stricture and morbid irritation in the canal. Hydrocele occasionally results from the irritation produced by loose accidental bodies in the tunica vaginalis, which are more frequently present than is generally supposed. In disturbed states of the circulation from disease of the heart, the tunica vaginalis is not so frequently the seat of dropsical effusion as the other serous membranes, with the exception of the arachnoid; but this is partly owing to the pressure exerted around the testis by the accumulation of fluid in the cells of the scrotum, and the relief to the spermatic vessels afforded by the œdema. In cases, however, of general anasarca, I have very frequently found slight effusion into the vaginal sac combined with œdema of the scrotum."

We have observed the combination of hydrocele with general dropsy rather more frequently, we think, than Mr. Curling appears to have done. Still it is not so common as, perhaps, might have been anticipated.

Mr. Curling is of opinion, and we quite agree with him, that, in spite of the disparaging remarks of Mr. Pott, the transparency of hydrocele is a very valuable sign. True, it is not always present, in consequence of thickening of the tunica vaginalis, &c.; but it is generally so, and affords the surgeon great assistance. Why should he neglect so ready and so valuable a means of diagnosis?

"In cases in which the parietes of the cyst are unusually thick, or the fluid is very dark-coloured, I have sometimes derived considerable assistance from using a wooden tube, about three quarters of an inch in diameter, open at both extremities. One end being placed against the swelling opposite the light, the surgeon, on looking through the other, can observe the transparency with great advantage. If a more convenient tube be not at hand, a roll of writing paper will answer the purpose. The growth of a hydrocele is occasionally attended with a good deal of local uneasiness, which has been ascribed to pressure on a nerve, or to the presence of accidental cartilages in the cyst. I have generally found, when pain exists, that the dropsical collection has originated in and been kept up by some disease of the testis. A hydrocele sometimes varies in size, being larger and more tense in the after part of the day than when the patient first rises in the morning. I have not exactly observed this change; but it has been so often mentioned to me by persons affected with hydrocele, that I entertain no doubt of the fact; and since the extent of surface afforded by the dilated tunica vaginalis is large, and the condition of the parts during day and night very different, such variations in size consequent upon alterations in the functions of secretion and absorption do not appear at all unlikely to occur. I have been informed of a case in which the change was so remarkable that the scrotum, which was full and tense when the patient retired to rest, became contracted and corrugated by the time he rose in the morning." 139.

In doubtful cases, we quite agree with Mr. Curling on the propriety of making an exploratory puncture with a grooved needle or a trocar. Mr. Curling observes:—

"I once met with an indolent tumour of small size in the scrotum of an old man, which was so irregular and uneven, felt so solid, and weighed so heavy, that it was impossible to determine exactly whether the swelling was occasioned by a morbid enlargement of the gland, a hœmatocele, or a hydrocele with the sac unusually thickened and indurated. The age of the patient was such as to put an operation out of the question. He subsequently died of disease of the chest; and, on examination, I found the tumour to consist of a hydrocele, the sac of which was cartilaginous and much thickened, and the contents a soft oleaginous kind of substance, consisting chiefly of cholesterine. The nature of such a swelling could only have been clearly ascertained by a puncture. The difficulty of the diagnosis, in cases of cartilaginous thickening of the tunica vaginalis, has been attested by Dupuytren. In a case of enlargement and induration of the left testicle, attended with lancinating pains in the groin and loins, and much emaciation, symptoms expressive of scirrhous disease, and unaccompanied with any sign indicative of hydrocele, or scrofulous or venereal disease, this distinguished surgeon, to avoid all chance of error, made an exploratory puncture. The result showed the prudence of this precaution; for, instead of scirrhus, the case was found to be a hydrocele, with cartilaginous thickening of the tunica vaginalis." 142.

It is, or rather it *was* a little while ago the fashion to abuse the grooved needle and its employment. They may dispense with it who never make mistakes, though such, in our opinion, are yet to make their appearance. Ordinary mortals had better use all the means in their power to avoid blunders, and this is one of the number.

But the grooved needle may deceive. A man had symptoms of suppuration in the tunica vaginalis testis. The grooved needle was introduced, and only serous fluid filled the hollow of it. The symptoms however grew worse, and an incision was made. It was found that pus had gravitated to the bottom of the tunic, the serum being supernatant. It was the latter that the grooved needle *tapped*.

Mr. Curling thus treats hydrocele in infants :—

"In these cases, all that is necessary in the way of treatment is a stimulating application, and support to the scrotum with a bandage. A lotion composed of an ounce of the hydro-chlorate of ammonia, four ounces of distilled vinegar, and six ounces of water, will generally cause the removal of the fluid. In many instances I have found it quickly disappear by occasionally painting the scrotum with the tincture of iodine. If the hydrocele does not disperse under this treatment in the course of two or three weeks, the tumour may be pricked with a cataract needle, which will allow the greater part of the fluid to drain away. This is the only operation that I ever found necessary in treating hydrocele in infants; and even acupuncture, which is a mild proceeding, and devoid of danger, is seldom required." 145.

Mr. Curling, speaking of tapping hydrocele, gives a hint on the state of the trocar. In selecting, he says, an instrument, the surgeon should see that the canula fits properly, and that its shoulder does not project too much; or else, after the point of the trocar has penetrated the cyst, the canula may hitch outside it, and, instead of entering the cavity, push the tunica vaginalis before it. In such a case, if the accident be not perceived in time, the testis or the back part of the cyst is very liable to be wounded. The trocar before being used should be thrust through a piece of wash-leather held tense, and unless it penetrates readily the instrument is unfit for use.

We would make another remark on the subject of trocars. In *injecting* a hydrocele, the surgeon should be careful that the *eye* of the canula is fairly within the tunica vaginalis. If it is not, some of the injection is apt to escape laterally through the eye, and to be forced into the cellular membrane.

Mr. Curling prefers the patient standing before him. So do we. But brave as he may seem, he is apt to grow faint and tumble forwards, which is awkward.

Mr. C. observes :—" The patient should be enjoined not to walk about much for the next twenty-four hours, and to abstain from active exercise for a day or two ; a precaution which is more especially necessary in individuals of an irritable or unhealthy constitution, or in advanced life. If this advice be neglected, acute inflammation of the tunica vaginalis is liable to succeed the operation. Some years ago I tapped the hydrocele of a healthy man fifty years of age, who, notwithstanding the caution I had given him, walked several miles the same afternoon ; the consequence was severe inflammation of the sac, followed by sloughing of the scrotum. After much suffering he recovered at the expiration of eight weeks, with the disease permanently cured. At a later period of life, if proper precautions be not taken, the palliative operation can scarcely be viewed as free from danger. Sir A. Cooper mentions two cases of persons in advanced age, who having taken a long walk after the operation, had inflammation and sloughing of the scrotum, which terminated fatally."

Suppuration in the tunic and diffuse inflammation of the cellular membrane of the scrotum are more frequent consequences of operations for hydrocele of all sorts, than is commonly imagined. We have seen several instances of both.

The following is Mr. Curling's mode of performing acupuncture of the hydrocele.

" I employ the common cataract needle, which I usually introduce in two or three different places, rotating the instrument between the finger and thumb to render the openings in the sac sufficiently patent. A little serum generally oozes out from the puncture in the skin in drops, or issues in a stream for a few seconds, and then ceases. In the course of a few hours the scrotal swelling becomes a good deal changed, and instead of a tense, smooth, and defined tumour, presents an œdematous tumefaction, with a soft, doughy, and inelastic feel. In large hydroceles the œdema extends to the integuments of the penis. The swelling thus produced takes from three days to a week gradually to disappear, the scrotum in favourable cases being left in its natural condition, without any excess of fluid either in its loose cellular tissue or in the sac of the tunica vaginalis. The operation may be repeated again and again as the fluid returns, on each occasion before the tumor has acquired the same size as on the preceding one, by which means the sac may sometimes be gradually reduced to its natural size." 154.

His estimate of the value of the operation is a candid one ;—" Acupuncture must be regarded, upon the whole, as a useful addition to our remedial measures for the treatment of hydrocele. It does not supersede the use of the trocar ; for the latter is scarcely more painful or less simple, and in careful hands is equally safe and free from hazard, whilst the immediate and certain relief which the trocar affords will always give it an advantage. Acupuncture, too, is ill adapted for cases of thickened sac ; and the chance of permanent benefit which it offers is too slight to add much to its value as a means of treatment. In very timid persons, in those of impaired constitutions, and in children, and in some other forms of hydrocele not yet described, acupuncture may be resorted to with benefit, and even preferred to the trocar. I am informed by Mr. Luke, that in the case of a gentleman who was about to proceed to a place in South America, where there would be no surgeon nearer his residence than 400 or 500 miles, he instructed his patient to perform this simple and harmless operation on himself."

Turning to the Radical Cure of Hydrocele, and speaking of Incision of the Sac, Mr. Curling states ;—

"My brother, Mr. H. Curling, of Ramsgate, informs me that when in Paris he witnessed several cases of hydrocele cured by incision by Jobert; but the treatment proved very severe, and confined the patients to bed for a long time. I have myself seen three cases of this disease attended with considerable thickening of the sac, which, after injections had failed, were successfully treated by incision; and certainly the consequences were less severe than the representations of Sharp and Pott would lead us to expect: but in these cases the tunica vaginalis was evidently less disposed to inflammation than usual." 157.

No doubt, however, it is a severe operation, and fit only for an exceptional one.

The *tent* has been more frequently employed than many practitioners may, possibly, suppose. Baron Larrey' plan was, after drawing off the fluid by means of a trocar, to pass a piece of gum elastic catheter through the canula into the interior of the tunica vaginalis, and to leave it there until sufficient inflammation to procure adhesion was excited. He speaks of this proceeding as being as mild as it is certain. "Such has not proved to be the case in other hands; and this, as well as the other forms of the tent, are in the present day rarely resorted to."

We are disposed to think more favourably of the tent than this. If introduced for a short time, say an hour, it is far from a severe remedy, and far from an unsuccessful one. A piece of gum catheter is the best.

Mr. Green has recently recommended the *seton*. His mode of performing the operation is nearly the same as that employed by Mr. Pott; but there is this important difference, that the seton is retained a much shorter period, the average time being twenty-four hours, though it will vary in different instances. In three of the eight cases treated on this plan which are reported, the re-introduction of the seton was necessary. In one case the cellular tissue of the scrotum suppurated, and in another an abscess formed in the vaginal membrane; both required to be punctured. In two instances the seton was obliged to be removed in a few hours, on account of the excessive pain which it produced. In the only three cases in which the seton operated mildly as well as successfully, one was cured in twenty-seven days, another in twenty-nine, and a third in about a fortnight. Certainly the results are not very encouraging

Mr. Curling adopts the following precautions after injecting a hydrocele. We should observe, by the way, that the injection he prefers is lime-water, of which he speaks in flattering terms. But to the after-treatment.

"My own experience does not incline me to rely much on the prudence of the patient in regulating the measures to be pursued after an operation. If, as generally happens, symptoms of inflammation arise in the course of a few hours, I usually recommend the use of a suspender to keep the testis supported, and direct the patient to remain in the recumbent position until the acute symptoms begin to subside. If these precautions be neglected, there is risk of more inflammation being excited than is necessary. Should no symptoms of inflammatory action be evinced in the course of eight or twelve hours, the patient should be encouraged to move about; and the testis may be handled, so as to occasion slight friction between the surfaces of the tunica vaginalis, in order to induce the requisite vascular excitement. If the swelling should become considerable, and the pain and constitutional disturbance be great, the activity of the inflammation must be moderated by leeches, saline purgatives, or tartar emetic, as in the treatment of acute orchitis. It sometimes happens that the inflammation goes on to suppuration, and occasions an abscess in the tunica vaginalis. I have never witnessed this; but when it occurs, an incision must be made through the integuments and sac, in order to permit the free escape of the pus. Granulation will then ensue, and the cavity of the tunica vaginalis will become completely and permanently obliterated. Sir B. Brodie remarks, that he has never known suppuration to occur after the operation by injection, except in West Indians, and in them only in three out of a great number of cases. In these cases the injected fluid was not made stronger than usual, but was even retained a shorter time—in one case only a single minute—and yet the inflammation was excessive; there was violent pain, and great constitutional disturbance. It may be well to bear this observation in mind, so that in operating on persons from warm climates the injections may be of a mild character." 171.

We apprehend that suppuration in the tunica vaginalis is more common than Mr. Curling states. Be that as it may, we are sure that some care is necessary after the operation, and that the off-hand directions of Sir Astley Cooper are anything but judicious or safe.

Mr. Curling very properly recommends a careful examination of the state of the testis prior to using an injection. He observes :—" A man was admitted into the London Hospital with a double hydrocele on purpose to undergo the operation for the radical cure. He had been suffering for some time previously from disease of the larynx, which increased soon after his admission, and caused suffocation and death. On examination of the testes, deposits of concrete pus were found in the substance of both the glands. In this case, had his state of health permitted of an operation, after removal of the fluid the diseased condition of the testes would probably have been detected, and injection, which could only have operated injuriously, would have been abandoned. The fluid effused around the diseased testis, by producing pressure, sometimes causes pain, and it may then be evacuated with benefit; but I need scarcely add, that to attempt the permanent removal of the hydrocele whilst the original disease remains unsubdued, would be both fruitless and hurtful. The disease producing the enlargement of the gland must be treated without reference to the effusion, and it will commonly be found that, as the affection of the testis subsides, the hydrocele likewise disappears."

Mr. Curling states, that even when the injection *is* unfortunately thrown into the cellular membrane of the scrotum, bad consequences do not always follow. He has known two instances in which there were no mischievous results.

*Iodine injections*, as most of our readers are aware, were first tried by Mr. Martin, of Calcutta, now practising in London. He used the tincture in the proportion of ʒij.—ʒvj. of water; injected only a small quantity; and instead of afterwards withdrawing the fluid, allowed it to remain in the sac to be removed by absorption. In a recent report of cases of hydrocele thus treated at the Native Hospital of Calcutta, it is stated that from the 9th of March, 1832, to 31st of December, 1839, 2393 cases were under treatment. Of these—

| | | |
|---|---|---|
| 1265 | were | Hindus, |
| 1076 | ,, | Mahomedans, |
| 52 | ,, | Christians. |
| 2393 | | |

And it appears that the failures were rather under one per cent.; a result which must be regarded as remarkably successful. The success and safety of iodine injections must be in a great measure attributed to only a small quantity of fluid having been thrown into the sac, so that risk of cellular effusion was avoided, and to the retention of the fluid insuring the excitement of sufficient inflammation to cure the disease.

European surgeons have tried this plan, some with more, some with less success, their report being, on the whole, favourable. Their real value, compared with other injections, is not yet determined.

With the following sensible remarks, Mr. Curling concludes the subject of simple hydrocels of the testis.

" A careful examination into the merits of the various modes of effecting the radical cure of hydrocele fully establishes the superiority of the treatment by injection. The great error formerly committed by surgeons in endeavouring to excite a high degree of inflammation arose from a mistaken view of the object to be attained; for not perceiving that the exundant secretion could be arrested by altering the action of the vessels of the part, they thought it necessary to obtain the obliteration of the natural cavity, which, moreover, they endeavoured to effect by producing suppurative inflammation of the membrane, instead of by the milder process of adhesion. In recent days, surgeons have sought to improve the treatment of hydrocele by reducing the amount of inflammation to the lowest possible standard, and have nearly fallen into the opposite error of suggesting plans too mild to be efficacious and sure. Injection has now been largely tried in this and other countries; and experience warrants us in asserting that though it is not an infallible remedy, of all the plans hitherto practised it combines the greatest number of advantages. The pain attending it is slight; its effects are mild, and at the same time tolerably sure; if properly performed, it is free from danger; and it frequently succeeds without altering the natural condition of the parts. I know it is a question whether the cure by adhesion, though less perfect than that in which the disposition merely of the vessels is changed, is not upon the whole preferable. In the latter there is a possibility, if not a probability, of a relapse at some future period, many of the causes conducing to hydrocele still remaining; whilst the inconvenience produced by an impediment to the free movements of the testis, in cases cured by adhesion, is regarded as too trivial to be any disadvantage. But, in the absence of data showing the degree to which the disease is liable to return after the cure without adhesion, I feel perfectly satisfied with such a result, and much prefer leaving a patient exposed to the doubtful chance of a relapse, than subjecting him to severer treatment in order to make sure of exciting sufficient inflammation to secure adhesion and obliteration of the sac. Injections, however, are not capable of effecting a cure in every case, nor are they adapted for every constitution." 180.

*Congenital Hydrocele.*

The only remark on this head that we need notice is the following:—

"There is rather a rare variety of congenital hydrocele, in which the testicle is retained in the abdomen or inguinal canal, whilst the peritoneum, prolonged for a short distance into the scrotum, forms the cyst containing the fluid, which is covered only by the integuments and superficial fascia. A hydrocele presenting the same characters as the congenital sometimes follows a late descent of the testicle, unaccompanied with a hernial descent. This is also a case of rare occurrence; but I once met with an instance in a lad eighteen years of age."

Mr. Curling very properly discountenances injecting congenital hydrocele. The fluid may, by pressure, be prevented from flowing into the abdomen, but that may not prevent the extension of the subsequent inflammation.

*Encysted Hydrocele of the Testis.*

The varieties of this are the following:—"The cyst is composed of a thin delicate serous membrane; it may be developed in three situations: 1. beneath that part of the tunica vaginalis investing the epididymis; 2. between the tunica vaginalis testis and tunica albuginea, which are thus separated from each other; 3. between the layers of the outer or loose portion of the tunica vaginalis. The first is by far the most common situation, the two latter being very rare. These cysts are analogous to the aqueous encysted tumors which are developed in the kidney and other parts, the fluid being of a similar nature, and differing from that of simple hydrocele in being perfectly limpid and colourless, and containing no or only a slight trace of albumen, so that it does not coagulate on the application of heat or by the action of acids."

Mr. Curling's account of Encysted Hydrocele of the Epididymis is good.

"Small serous cysts," he says, "not larger than a pea, and even smaller, frequently exist immediately beneath the tunica vaginalis covering the head of the epididymis, in which they produce a slight depression. In several instances I have found as many as five or six perfectly distinct cysts connected with this part. Sometimes one or two small cysts of this kind are so embedded in the substance of the epididymis, that they cannot be recognised without dissection. Though these minute cysts generally contain a limpid serum, I have found them filled with fluid of a milky hue, and I have even observed matter like pus tinged with blood. These accidental cysts, developed in the upper part of the epididymis, sometimes project the tunica vaginalis before them until they become so far separated from the part where they were originally formed, as to be attached only by a narrow peduncle formed by the contracted tunica vaginalis. Such is the mode of development of those small pendulous pedunculated cysts containing an aqueous fluid often found hanging from the head of the epididymis, which were erroneously supposed by Morgagni to be hydatids. I have on many occasions observed them in the different stages of their production. Thus I have seen a pedunculated cyst attached at one part, whilst close to it there was a serous cyst precisely similar embedded in the substance of the epididymis. In other instances I have found the cyst very prominent, but still connected by a broad attachment of the tunica vaginalis reflected over it, the membrane not having as yet contracted to form the narrow neck. In all these cases the prolongation of the tunica vaginalis investing the cyst could always be demonstrated by a little cautious dissection, and between this membrane and the cyst some minute red blood-vessels were generally seen ramifying. These pedunculated cysts never acquire a large size; I have seldom found them to exceed that of a currant. From the exposed situation of the testis, they are liable to be ruptured, the vestiges of them consisting of fimbriated folds of membrane; but this is not a common occurrence. I have seen the delicate peduncle by which the cyst was attached as long as three quarters of an inch.

So common are small serous cysts connected with the epididymis in the various states and stages I have described, that no one can examine many testes without finding them. Now when one or more of these cysts, instead of becoming pedunculated, enlarge so as to form an evident tumor in the scrotum, they constitute a form of hydrocele called, from its original seat, an *encysted hydrocele of the epididymis.* I have observed this description of hydrocele in all its various modifications, from the enlargement simply of a single cyst to the complication occasioned by the varied dilatation of several of them. In this form of hydrocele the epididymis becomes flattened, and is displaced to one side, whilst the testis is found either in front of the cyst or cysts, or at the bottom of them. It is sometimes at the side, but very rarely indeed at the posterior part of the swelling."

"The tumor is generally of smaller size than a simple hydrocele, the fluid seldom exceeding three or four ounces in quantity. In a case, however, which I saw with Mr. Crowdy of Brixton, as much as twenty ounces were removed from a single cyst. When the hydrocele is composed of several cysts, they are seldom of large size, but form a cluster more or less complicated and irregular, according to their number." 188.

The cysts are liable to inflammation and its consequences, an albuminous state of the fluid, lymph, &c. in the cyst. Or this may contain blood, and form a variety of hæmatocele.

2. *Hydrocele between the Tunica Vaginalis Testis and the Tunica Albuginea.* The cyst is usually single and small. It is rare. Mr. Curling has seen one instance of it. Sir B. Brodie has described one.

3. "In examining a healthy testis I once found six or seven small serous cysts, about the size of currants, studding the surface of the loose portion of the tunica vaginalis. Two of them were situated in a part of the membrane extending up the cord. They projected internally, and contained a transparent fluid. I have since seen a similar kind of cyst, the size of a large pea, in the same portion of the tunica vaginalis. Accidental serous cysts have also been observed in the sac of a simple hydrocele, and a preparation of this kind is contained in the museum of the College of Surgeons. If a cyst thus situated were to increase to any size, it would constitute a swelling which might be appropriately termed an *encysted hydrocele of the tunica vaginalis.*" 190.

*Diagnosis.*—This is not always, perhaps, practicable. Encysted hydrocele of the testis may, however, be said to be distinguishable from simple hydrocele by the different position of the gland, which is generally found in front or at one side of the tumor; by the smaller size of the swelling; and by the limpid and colourless character of the fluid evacuated. When the hydrocele consists of two or more cysts, fluctuation and transparency are also less distinct than in simple hydrocele. The common experimentum crucis will be the puncture.

*Treatment.*—For palliation, when the cyst attains any size, acupuncture or the trocar—when the radical treatment is *necessary*, the seton, injections not answering so well as in simple hydrocele.

There is so much in this volume to engage attention, that we shall return to it in our next number, and complete our account of its contents. In the mean time we can confidently recommend it to our readers.

